# Examining Interprofessional teams structures and processes in the implementation of a primary care intervention (Health TAPESTRY) for older adults using normalization process theory

**DOI:** 10.1186/s12875-020-01131-y

**Published:** 2020-04-15

**Authors:** Ruta Valaitis, Laura Cleghorn, Lisa Dolovich, Gina Agarwal, Jessica Gaber, Derelie Mangin, Doug Oliver, Fiona Parascandalo, Jenny Ploeg, Cathy Risdon

**Affiliations:** 1 Aging Community and Health Research Unit, School of Nursing, McMaster University, HSC 3N25, 1280 Main Street West, McMaster University, Hamilton, ON L8S4K1 Canada; 2 Department of Family Medicine, McMaster University, David Braley Health Sciences Centre, 100 Main Street West, 5th floor, Hamilton, ON L8P 1H6 Canada

**Keywords:** Primary care, Interprofessional team, Volunteers, Implementation, Normalization process theory, Older adults

## Abstract

**Background:**

Many countries are engaged in primary care reforms to support older adults who are living longer in the community. Health Teams Advancing Patient Experience: Strengthening Quality [Health TAPESTRY] is a primary care intervention aimed at supporting older adults that involves trained volunteers, interprofessional teams, technology, and system navigation. This paper examines implementation of Health TAPESTRY in relation to interprofessional teamwork including volunteers.

**Methods:**

This study applied Normalization Process Theory (NPT) and used a descriptive qualitative approach [1] embedded in a mixed-methods, pragmatic randomized controlled trial. It was situated in two primary care practice sites in a large urban setting in Ontario, Canada. Focus groups and interviews were conducted with primary care providers, clinical managers, administrative assistants, volunteers, and a volunteer coordinator. Data was collected at 4 months (June–July 2015) and 12 months (February–March 2016) after intervention start-up. Patients were interviewed at the end of the six-month intervention. Field notes were taken at weekly huddle meetings.

**Results:**

Overall, 84 participants were included in 17 focus groups and 13 interviews; 24 field notes were collected. Themes were organized under four NPT constructs of implementation: 1) **Coherence**- (making sense/understanding of the program’s purpose/value) generating comprehensive assessments of older adults; strengthening health promotion, disease prevention, and self-management; enhancing patient-focused care; strengthening interprofessional care delivery; improving coordination of health and community services. 2) **Cognitive Participation**- (enrolment/buy-in) tackling new ways of working; attaining role clarity. 3) **Collective Action**- (enactment/operationalizing) changing team processes; reconfiguring resources. 4) **Reflective Monitoring**- (appraisal) improving teamwork and collaboration; reconfiguring roles and processes.

**Conclusions:**

This study contributes key strategies for effective implementation of interventions involving interprofessional primary care teams. Findings indicate that regular communication among all team members, the development of procedures and/or protocols to support team processes, and ongoing review and feedback are critical to implementation of innovations involving primary care teams.

**Trial registration:**

ClinicalTrials.gov, no. NCT02283723 November 5, 2014. Prospectively registered.

## Background

Many nations are arguing for the need to increase comprehensive and coordinated primary care services in light of an aging population that is living longer and managing multiple chronic conditions [2]. Primary care is widely regarded as the locus for access and optimization of care for older adults, and many countries are engaged in primary care reforms to meet populations’ needs [3]. Despite several decades of reform aimed at increasing access to and coordination of primary health care services, the Canadian health care system remains fragmented and primary care is largely detached from secondary and community care [4]. Older adults also face barriers in accessing health and social care systems to address their needs resulting in higher use of emergency rooms and hospitalizations [5]. Furthermore, the care that they receive from primary care is reactive rather than proactive, and joint goal setting, action planning, and care planning rarely occurs [6, 7]. Given that older adults experience multiple co-morbidities [8, 9] they can benefit from person-focused, wellness-oriented, tailored approaches to care [10] to support them to age in place. Interventions to address older adults’ needs as well as manage chronic diseases typically require the adoption of models of care that support interprofessional team- and community-based primary care for older adults [11, 12]. This paper examines the implementation of an innovative team-based primary care model to address the needs of this population.

A primary care intervention was developed through engagement of interprofessional primary care team members, volunteers, community service providers, and older adults [13, 14]. Health Teams Advancing Patient Experience: Strengthening Quality [Health TAPESTRY (HT)] is an innovative, coordinated approach to care which seeks to build on existing healthcare system strengths while attempting to address its challenges [15]. HT is centred on meeting people’s health goals and needs to help them live at home healthier and longer. It is argued that complex interventions are needed with multiple interacting components to produce change [16]. As such, HT is comprised of four components: 1) interprofessional teams, 2) trained community volunteers, 3) e-health technologies, and 4) increased linkages between primary care and community organizations.

Pairs of volunteers visit older adults at home, build relationships, and act as connectors between the patient and their primary care team. Volunteers receive training related to role expectations and activities which can help to ensure that they are not assuming a health professional role. Roles included forming relationships with clients, gathering patient information around health needs and health-related goals, assisting clients to set up a personal health record, sharing community resource information and providing motivational support. Training is provided to support each of these roles [14]. The volunteer pair consists of an older adult and a younger adult, who is often a university student. This team collects data on older adults’ health needs and goals using questionnaires on a tablet (via an online application). The surveys assess mobility, physical activity, nutrition, memory, medication, and also include open-ended questions about life and health goals.

HT reports completed by the volunteers are sent electronically to the patients’ electronic medical record. Interprofessional primary care teams meet in weekly interprofessional “huddles” (small team-level communication) [17] to triage HT volunteer reports and support care coordination. Huddle teams of 5 to 6 providers engage in system navigation tasks as they facilitate older adults’ access to primary care and community-based programs and services. Communication occurs between the most responsible physician (MRP) and other clinicians beyond the huddle as needed. Huddles are not part of usual practice. However, interprofessional teams do participate in informal consultations among team members, and all primary care team members attend monthly academic rounds.

A randomized controlled trial was conducted to evaluate implementation and effects of the HT intervention [15]. Guidance provided by the Medical Research Council indicates that it is important to understand both outcomes and processes of any new complex intervention [16, 18]. Having a theoretical understanding is needed to explain how an intervention might be causing change, since effects can be influenced by implementation failure or success [16]. Furthermore, without a deep understanding of implementation processes, effective interventions have small likelihood of being applied into real world settings [19].

There is growing interest in applying theories and frameworks to explore implementation [20]. We used Normalization Process Theory (NPT) to examine the implementation of HT’s four components [21]. This theory has been widely used to explore implementation of health care interventions, specifically in primary care contexts [19, 22, 23]. As a formal mid-range theory it is particularly appropriate for this study. It was developed to address observed challenges in integrating new interventions and reorganizing care delivery in health care settings [21]. NPT posits that complex interventions are implemented via processes and integrated via structures in a professional and organizational context. Normalization is a result of the work people both individually and collectively undertake to implement and embed an innovation. NPT consists of four constructs [21]. **Coherence** (i.e., sense making) refers to how everyone individually and collectively understands the intervention, its purpose, and potential value. It also includes how individuals and the team see the intervention as differing from usual care delivery. **Cognitive participation** (i.e., enrolment) refers to individual and leadership buy-in of the intervention and agreement to work with it. **Collective action** (i.e., enactment) is operational; people have the needed resources to undertake new practices, and everyone knows who is doing what. **Reflexive monitoring** (i.e., appraisal) includes feedback gathered on outcomes and impacts of the intervention, which can reinforce its continued application and adaptation.

A 2017 integrative review applied NPT to explore barriers and enablers in implementing interdisciplinary team working in primary care [23]. The review showed that there was a paucity of research that examined all four NPT core constructs, other than collective action. It also showed that most implementation studies look at a few team members without considering the full team [23]. The current study, therefore, aims to examine the implementation process of the HT intervention in relation to teamwork including all team members (providers, clinical managers, and volunteers) and patients. Results may inform others implementing complex interventions involving primary care teams.

### Research questions

The primary care team was conceptualized as consisting of: 1) small interprofessional huddle teams, who received and reviewed the HT patient reports, 2) primary care team members outside the huddle teams, and 3) trained HT volunteers. The research questions were:

1. What are the perceptions of patients, volunteers and health care providers on the implementation of HT in relation to the work of interprofessional teams?
2. How has HT implementation affected existing structures and processes of primary care with the teams?

## Methods

### Design

This study used a descriptive qualitative approach [1] to explore implementation processes of HT. The study was embedded in a mixed-methods, pragmatic randomized controlled trial [15].

### Setting

This study was situated in two primary care practice sites in a large urban setting in Ontario, Canada. McMaster Family Health Team, is a multidisciplinary team providing 7-day-a-week care, supported by an electronic medical record. Physicians are remunerated through blended capitation and affiliated with a partially provincially funded (salaried) interprofessional team [24]. The family health team has approximately 37,000 patients, 21 family physicians (full-time equivalent), 28 family medicine residents, 8.5 nurse practitioners, 5.5 registered practical nurses, and 26 full- or part-time other health care professionals (occupational therapists, physiotherapists, social workers, dietitians, pharmacists, psychologist, system navigators, physician assistants, a lactation consultant, and a chiropodist).

### Sample and recruitment

Participants included: health care providers, clinical managers, administrative assistants, HT volunteers, and a HT volunteer coordinator. Invitations to participate were emailed to all family health team members of the weekly interprofessional “huddle” teams, clinic managers, nurse practitioners, as well as physicians and medical residents at each site who had 10 or more patients participating in the Health TAPESTRY (HT) program. All HT volunteers and the coordinator were invited via email to participate in focus groups while the volunteer coordinator was invited to an individual interview. Initially, all patients who completed the HT intervention were invited to participate in an interview, with the goal of selecting 30 participants equally distributed by gender, clinic site, and across 2 age groups (70–79 and 80+). Selected HT patients were recruited by phone. We found this was resulting in a healthier sample of older adults based on their HT survey results, therefore, we selected from individuals identified with greater health needs based on their survey responses.

### Data collection

Data were collected at two time points: 4 months (June and July 2015) following the start of the intervention and 12 months (February and March 2016) for all participants except for HT patients who were interviewed at the end of the 6 month intervention (November 2015 to February 2016). Phone interviews averaging 30 to 45 min were held with clinic managers, the volunteer coordinator, and physicians who were not part of the huddle team or could not attend a focus group. One-hour focus groups were conducted with huddle teams, physicians and medical residents at their workplace. Volunteer focus groups were held separately with older adult and student volunteers in an accessible room in the community. One-on-one interviews were held with patients in person at clinic or on the phone.

Data collection was completed by a nursing researcher and Department of Family Medicine research staff experienced in qualitative methods (LC, RV, JG, FP, MB, NF, DJ). All were female. A few providers were known to some research staff since they had interacted through previous studies. Participants were informed that the study was focused on learning about their experiences implementing the intervention, their thoughts on how well the program was working, impacts and outcomes, and program sustainability. Interview guides (available on request) were informed by NPT’s core constructs and questions were altered slightly based on participant type and divided into sections in keeping with the topics noted above. Patient interview questions focused on their understanding of HT, their experiences with the program, and perceived impacts on communication with the team, coordination of care, and sustainability. Participants received a $25 CAD gift card as a token of appreciation. Light refreshments were provided at focus groups. All interviews and focus groups were digitally recorded and transcribed verbatim.

In addition, a researcher (LC) took field notes during huddle meetings to track changes in the interprofessional team structure and processes and noted challenges and enablers in implementation. Any significant challenges were shared with the implementation team to provide them an opportunity to adjust their processes.

Results are organized according to NPT constructs. Quotes are used to support themes marked by the source as follows: Huddle team member = [Hud-1], [Hud-2], etc.; primary care team members other than the huddle team = [PC-1]; Volunteer = [Vol-1], etc.; and patient = [Pat-1], etcetera.

### Analysis

Authors RV and LC have extensive experience in qualitative analysis. The coding structure was created based on interview questions and the intervention components (interprofessional teams [huddles and the larger primary care team]; volunteers; technology; and system navigation). Once the initial structure was developed, it was discussed amongst coders (FP, JG, NF) who then individually and inductively coded transcripts using NVivo Version 10 software [25]. RV and LC supervised coding of the initial transcripts. They met over 4 to 5 half-day meetings held monthly during the active coding period to discuss meanings of codes and ensure consistency. After all transcripts were coded, RV and LC checked random selections of transcripts. Using constant comparison, they further refined the coding structure and developed themes and their elements (sub-themes). In the last phase of analysis, RV and LC met over multiple meetings to move themes and/or sub-themes under the constructs of NPT [21] which served as sensitizing concepts to guide the final analysis [26, 27]. The full research team met on 3 occasions to review the coding structure and reach consensus on final themes and their elements.

## Results

### Participant characteristics

Overall, 96 participants were involved in 17 focus groups and 13 interviews conducted over the two time points- 4 and 12 months. Table 1 shows the numbers of participants in focus groups or interviews including primary care team providers (*n* = 29), health care managers (*n* = 2), administrative support (*n* = 2), volunteers (*n* = 30), and the volunteer coordinator. There were more female than male volunteers. Thirty-two patient interviews were conducted at the end of the intervention. Most were aged 70–79 years.

**Table 1:** Total Number of Participants in Focus Groups or Interviews

Participants	Total n (%)	Focus Group n	Interview n
**Health Care Providers**	29 (30.2)		
Type			
Allied Health (Dietitian, Occupational Therapist, Physiotherapist, Pharmacist, System Navigator)	10 (34.5)	10	
Nurse (NP, RPN)	7 (24.1)	7	
Family Physician/Medical Resident	11 (37.9)	7	4
Psychologist	1 (3.4)	1	
**Health Care Managers**	2 (2.1)		2
**Administrative Support**	2 (2.1)	2	
**Volunteer Coordinator**	1 (1.0)		1
**Volunteers**	30 (31.3)	30	
Type			
Student	16 (53.3)		
Mature	14 (46.7)		
Gender			
Female	21 (70.0)		
Male	9 (30.0)		
**Patients**	32 (33.3)		32
Age			
70–79 years	19 (59.4)		
80 years +	13 (40.6)		
Gender			
Female	16 (50.0)		
Male	16 (50.0)		
**Total**	96 (100)	**57**	**39**

### NPT constructs

Within each construct, themes are highlighted with their corresponding elements that describe each theme (see Table 2).

**Table 2:** Normalization Process Theory Constructs, Themes and Elements

Themes	Elements of the theme
**Construct #1: Coherence (sense-making, purpose of the intervention)**	
Generating comprehensive assessments of older adults	1. Better information about client’s needs, goals, risks, wants obtained through volunteer visits 2. Data collection screening processes improved 3. New patient information generated to support more comprehensive care and follow up
Strengthening health promotion, disease prevention, and self-management for aging at home	1. Care shifting to be more proactive and focused on health promotion and disease prevention 2. Seniors supported to age at home 3. Improvements in self-management 4. Enhancements in health education
Enhancing patient-focused care	1. Caring and open relationship with patients and volunteers as confidantes 2. Patient engagement in care enhanced wherein patients are more connected and have a voice 3. Patients feel valued and cared for by clinic staff
Strengthening interprofessional care delivery	1. Strengthened team-based approach to care 2. Role of volunteers in supporting primary health care explored
Improving coordination of health and community services	1. Knowledge of community resources by patients and team increased 2. Improvements to access to community-based resources
**Construct #2: Cognitive Participation (buy-in, engagement)**	
Tackling new ways of working	1. Huddle teams experience the biggest changes in ways of working, while those not in the huddle teams experience the least 2. Huddle coordinator facilitates MDs, residents and multi-disciplinary team to contribute new patient information to huddle and coordinate care 3. Volunteer role accepted by patients as part of the health care team, but could be misinterpreted as health professionals by patients
Attaining role clarity	1. Challenges for primary care providers outside of huddles (i.e., MDs, residents) to understand their roles in relation to the huddle team, HT reports and alerts and follow up with patients 2. Lack of clarity by volunteers regarding their role with patients (e.g., advice giving) (for some) 3. Huddle team members learn one another’s roles and perceive benefits through increased teamwork and collaboration
**Construct #3: Collective Action (operations, resources, enactment)**	
Changing Team Processes	1. Improved care coordination and case management process changes related to the new huddle team structure (e.g., ‘chart and chat’, follow up, case conferences, and referrals) 2. Improved flow, content and sources of patient information changes 3. New proactive approaches for the care of aging developed (e.g., prevention and promotion) 4. Some challenges exist in relation to primary care follow up and potential loss to follow up 5. Communication challenges existed with team members outside the huddle re. action plans
Reconfiguring Resources	1. Shifts in structure and increases in workload for primary care huddle team 2. Clinic human resources took time to get organized for best use, (i.e., providers understanding their own role and part in the process)
**Construct #4: Reflexive Monitoring (appraisal, evaluation, feedback)**	
Improving teamwork and collaboration	1. A more effective model for collaboration is now embedded in the clinic 2. Team communication and understanding of one other’s roles improved 3. Interprofessional team huddle perceived to be valuable and worth maintaining 4. Patients experienced satisfaction with healthcare team and system
Reconfiguring roles and processes	1. Changes to flow of information, patient referral and follow up 2. Clarification of roles of huddle team members and wider primary care team 3. Explore efficiencies and sustainability of the program

#### **Construct 1:** coherence - sense making

Coherence refers to the sense-making work that people do individually and collectively when they are faced with implementing a new practice or intervention. Here, we consider how the actors understood HT and aims and benefits of the new model. In addition, we explore if and how the work differs from the usual way of working. Five themes emerged in relation to coherence as follows.

1. Generating comprehensive assessments of older adults:

Participants noted that one of the main goals of HT was for primary care to generate improved screening and more comprehensive assessments of older adults including their needs, goals and risks. A volunteer explained how their home visit, and the questions they ask, differed from standard care:

*… a lot of the questions we ask aren’t really questions that a [family physician] would necessarily ask their patient, depending on why they happened to be there on a particular day. [ … ] most people go to the doctor for a reason and you have 15 or 20 minutes to get that reason sorted out and so they don’t really ask well, “What’s your diet like*” [Vol-8]

2) Strengthening health promotion, disease prevention, and self-management for aging at home

This theme was related to shifting to more proactive care focused on health promotion and prevention to support older adults to age at home. A member of the interprofessional huddle team noted:

*The theoretical value and benefits are that people would be able to take some proactive steps and have some assistance in improving their function and meeting the goals that they have to stay socially connected and to stay mobile and to be prepared for the aging process.*. [PC-4].

HT was also seen as having the aim of improving self-management and enhancing health education.

3) Enhancing patient-focused care

Participants perceived that HT would support patient-focused care through the development of open relationships with patients and volunteers as confidants on the team. One volunteer described the benefit they perceived clients had from having a volunteer in the home that helped build relationships and encouraged clients’ to be open to share:

*… it also gives them a person that they feel comfortable talking to. So, in addition to the time, just having that space to talk. But also having a person that they feel like, ‘we are on the same level. So, I can tell them any problems that I won’t want to tell my doctor because he’ll judge me’...* [Vol-6].

Participants also felt that there would be more patient engagement in their care, with patients being well connected to the primary care team, with a greater voice in their care. Finally, HT would help patients feel more valued and cared for by clinic staff. A huddle member explained:

*I think a lot of the reports that we get feedback from them, when we see them in follow-up to this and they’re like, this is really, really great, ‘I didn’t realize that you guys cared so much …* [Hud-13].

4) Strengthening interprofessional care delivery

Participants perceived that HT would strengthen team-based approaches to care by creating a one note for the client record that indicated the plan of care that was based on team input.

*I think that the communication piece with the other providers is something that is better with this structure. So, these patients wouldn’t have had that type of communication where there’s that one note with all of us sort of saying, ‘this is the plan that we are going to do’.* [Hud-4].

Another team member explained that although usual care involved other professionals, it wasn’t inclusive of all interprofessional staff and they were not always ‘*at the table*’. One participant explained the value of having various professionals at the table to better see how interprofessional team members can contribute to care:

*I’m glad to see that it is now, because you can make an assumption about other people’s roles and skills. But what I have discovered is those people need to be at the table because they see things within their roles and skills that others may have presumed they could but can’t*. [PC-2].

Another benefit was the role and additional time spent by the volunteer as an extension of the primary care team as described by a clinician:

*... we often don’t have enough time to spend with them, and that’s what we need sometimes, just to learn about their lives and what are their main things that are important to them and where can we sort of provide the support to help*. [PC-22]

5) Improving coordination of health and community services

Two elements of improving coordination describe the final theme in coherence; the first was that patients and team members increased their knowledge of community resources:

*I think they’re learning about community resources that you would know about, about [exercise program], the [online information portal]. […]That’s all new for a lot of these people.* [Vol-10].

Second, a few perceived that HT was a way to improve access to community-based resources:

*… we really link some patients with community resources out there that they normally wouldn’t have been linked to through their family doctor’s office; that they would have had to go out and find those community resources on their own...* [Hud-8].

Others spoke about increased integration with the community by locating people within their communities.

#### Construct 2: cognitive participation - enrolment

Cognitive participation is the work that people do together, to build and sustain a community of practice around a complex intervention. It addresses how the work is driving new practices forward, how individuals re-think their roles and relationships with others, how they re-organize themselves to do the work, and how actions and procedures are created and sustained. This construct had two themes.

1. Tackling new ways of working

The new huddle teams began working with clinic staff who they had not normally worked with in the past, such as the dietitian, occupational therapist, and pharmacist.

*I think that even though we have more people at the table, it’s become much more focused and the conversation that I have seen is much richer. So, I think it’s evolved to a nice point where I see it being a sustainable model for having interprofessional discussions*. [PC-2].

The role of the huddle coordinator was viewed as essential by the team, although it took time to develop. The huddle coordinator was the case manager to determine the best team member/s to follow up on patient issues, work with the team to contribute patient information, and arrange follow up care. The coordinator was:

*… kind of the gatekeeper of the reports, [who would] loop around to make sure everything has been done.* [Hud-8].

In addition, huddle team members, including physicians, contributed new information about patients, picking up pieces that others had missed, for a more holistic assessment and plan.

*I think that [the] occupational therapist just has a sensitivity and sort of receptors for a very different group of issues with our patients than the doctors and the nurses do*. [PC-4].

However, some clinicians who were not huddle team members did not experience HT in the same way. One huddle team member reflected that some clinicians got to know their patients very well without HT and therefore the program did not really seem to be different for them:

*The issues that are coming up are probably known issues, first of all; maybe things that [the physicians] have already addressed.* [PC-22].

On the other hand, some physicians contributed to care planning with the team for their patients. Volunteers were seen as adding value and contributing to the health care team; however, patients weren’t always clear about volunteers’ roles. Some perceived them to be health professionals.

*I am sure they assume that you know more than you do as a volunteer*. [Vol-1].

  2) Role clarity.

Clinicians outside the huddle team were unclear about: their roles in relation to HT huddle teams, how to handle HT reports that focused on alerts rather than the full report, and their role in patient follow up. Physicians were unclear about their roles versus those of the huddle team members in following up with HT patients. One physician explained:

*I didn’t get any information from the multi-disciplinary team about some of my patients and then I don’t know what to actually do about that. Actually, I do, but I don’t know if any of them will call the patient, book an appointment for me. Do I have to do that myself? So, this is the part that’s for me, personally, a disconnect.* [PC-16].

Generally, volunteers were comfortable in their roles and working in pairs, understanding that they were to collect data, be a liaison to the primary care team, and support goal identification. They defined their role as the front line of the project, non-experts, flexible, and committed information providers. Some volunteers, however, lacked clarity regarding their role in relation to giving advice to patients.

*There’s also some discomfort knowing how much advice to give and what sort of.*

*advice you should be giving — given that we are not professionals or health care providers.* [Vol-6].

For huddle team members, clarity in their own and other’s roles became clearer through continued teamwork and collaboration.

#### Construct 3: collective action or enactment

Collective action is the operational work that people do to enact a set of practices. What were they actually doing and who was doing what? This is about interacting with the intervention, establishing accountability and confidence in a new system, and how resources are allocated to support its ongoing implementation.

1. Changes to Team Processes

In this theme, care coordination processes improved through the implementation of team huddles and better interprofessional team partnering to: better understand patients’ needs and goals, conduct more follow ups, make referrals, ‘chart and chat’ with team members who know the patient best, and use the right provider for the right care at the right time. As one huddle team member described:

*Just the conversations that we have, every report … when we go through them, the things that it triggers for the different people and the follow up that happens. And we have our group discussion and we each take away pieces if needed, or, we do some stuff together. [Hud-3] and I will do things together when we get to it or the psychologist and I. And there’s a lot of partnering and pairing and sharing of stuff*. [Hud-1].

A few patients noted changes in how care was provided, although they could not necessarily attribute this to HT. A patient noted:

*Well, this one lady came and she talked to me about all the exercises and stuff like that. I don’t know if [HT] included that; I have no idea*. [Patl-148].

The flow and sources of patient information also improved. More comprehensive patient information collected from different sources (e.g., the volunteers) highlighted patient information in new ways that raised team discussion. A manager shared that:

*It has highlighted the risk of things that are higher, like malnutrition, the lack of exercise, memory issues. […*] *I think they have just really brought them to the forefront, which is actually a good thing because now there can be a more organized response*. [PC-1].

In comparison to usual brief clinic appointments, volunteers and health care providers appreciated that patients had more interaction with the team through volunteer home visits, as well as opportunities to identify potential gaps or pick up on things potentially missed by the clinic staff. The flow of information also changed in that the huddle team had the new task of determining how to triage and respond to HT reports and the patient issues that surfaced. There was a sense that teams were using new proactive approaches for the care of aging which focused on preventing crises through referrals and consultations. One MRP noted,

“*… in our clinic the whole process of being sort of proactive and of case discussions around these particular sorts of people was brand new*.” [PC-4].

Compared to usual care, many patients expressed that they had follow up after a volunteer visit from the MRP or various allied health providers, (occupational therapists, physiotherapists, pharmacists, or multiple providers). Other patients, however, expressed discontent because there was no follow up visit or contact from the volunteer or clinic and some were offered follow up but expressed disinterest. Although many patients reported being connected to a community service or resource by the team, not all were able or interested in using the service. Patients also noted that they were unclear about how information was shared between volunteers and clinicians.

There were some communication challenges between huddle and clinicians outside the huddle. Action plans were not clearly shared beyond huddle teams. Some residents were unaware of HT. As noted by a primary care team member outside the huddle:

*Every once in a while, I get a message from somebody that’s sort of, you know, copied on the [HT] report say, ‘oh, this is what we’re doing’. I have had sometimes, a message saying, ‘Don’t worry about this, ignore this’, or something of that nature, but I would agree with (PC-19), to me it’s a complete black box.* [PC-26].

This was rectified near the end of the intervention by having a designated champion from the huddle team assigned to each patient to support communication with other clinicians beyond the huddle team.

**Fig. 1:**
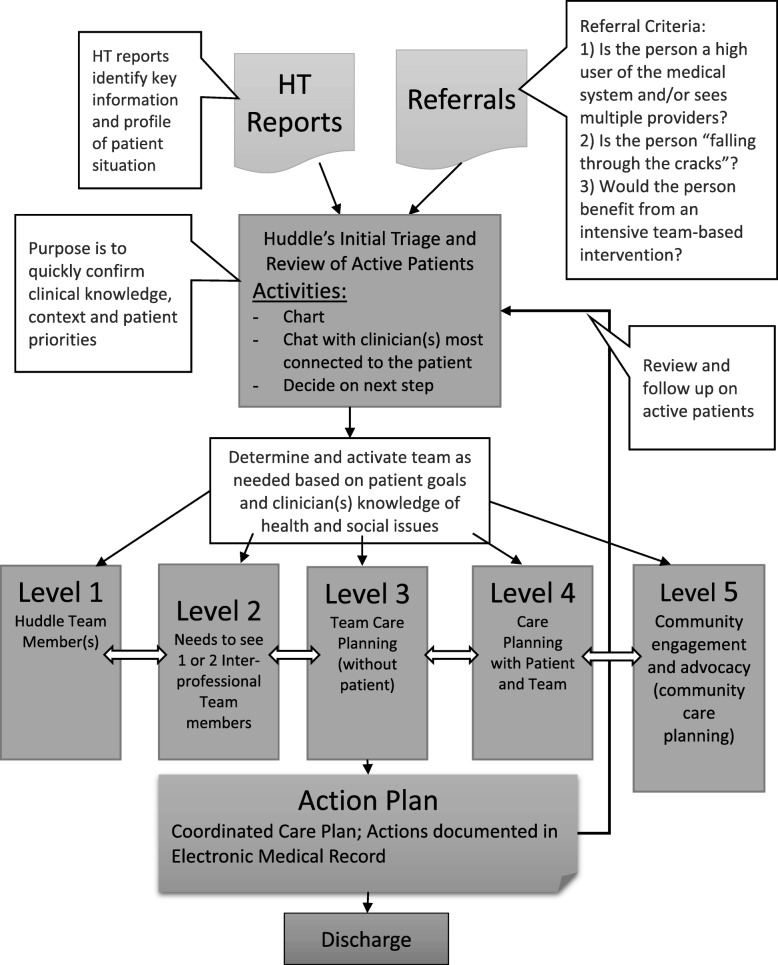
Interprofessional Team Huddle and Report Triage Process

Fig. 1 illustrates the decision-path that was created in the early stages of HT for care triaging patients by the interprofessional huddle team to coordinate care.

  2. Reconfiguring Resources:

This theme referred to structure changes, shifts in workload, and the time that it took to get clinic resources organized to ensure effectiveness. The huddle team structure changed in terms of time, space and composition. Clinic staff outside the huddles and huddle teams particularly noted changes in team make up.

*We have players at the table we didn’t even have three years ago, like physiotherapy, for example, or chiropody possibly. Or we have added to the team as well, which has also changed the dynamic.* [PC-6].

Another huddle member explained the way teamwork differs from usual practice in the HT program:

*There’s never six of us in a room together focusing on a list of certain patients.At this site, that’s the biggest difference*. [Hud-8].

Concerns existed related to new work generated by the intervention, such as booking appointments, making referrals, and huddle meetings, which took time away from more urgent cases and increased wait lists for the clinicians.

*Unfortunately for time reasons, it has taken away from seeing some of the other patients that I thought would have been more needy or more requiring the house calls because I’m here now dealing with some of these healthier guys*. [PC-12].

It took time to organize the resources in the best way possible to support the program. For example, there was a need to determine a huddle lead and establish administrative support to take on the non-clinical roles such as booking meetings, which was seen as a burden, and making follow up phone calls to patients. Time was also needed for interprofessional collaboration.

*I still feel that to get the collaborative, people all have to have a stake in the game and all have to like look at the chart and look at the report and that just takes time. [...] I think having this facilitator who would help facilitate which charts the Allied Health needs to read through. I think that job has the richness of the Allied Health team, so it takes time. And Allied Health rounds, they all take time.”* [PC-8].

#### Construct 4: reflexive monitoring or appraisal

Reflexive monitoring is work that people do to assess and understand the ways that a new set of practices affect them and others around them (e.g., feedback and monitoring). What did they think were good outcomes and impacts? How were they judging this? Appraising the intervention can lead to attempts to redefine procedures or modify intervention practices.

1. Improving teamwork and collaboration

Implementation of HT was perceived to have a positive effect on teamwork and clinic collaboration. It enhanced communication to inform patients’ care plans through face-to-face conversation supported by robust charting in the electronic medical record. Clinic participants also reported that the intervention provided a model for improved interprofessional team collaboration, resulting in a greater understanding of clinicians’ roles and skill sets. Huddles were viewed as worth creating and maintaining, both for interprofessional team and patient benefits. Participants identified that the majority of patients were satisfied with the HT interprofessional approach to primary care. Overall, they deemed the intervention worthwhile and sustainable. Changes to interprofessional teamwork structure and processes were considered possible to sustain, suggesting that normalization had occurred.

*For me, a neat thing is the inter-professional team and seeing what everybody else has to offer or bring to the table, looking at a patient and having a fresh set of eyes look at a patient’s chart from different angles. […*] *As a clinician in the team, seeing what everybody else does, and appreciating that; how much added benefit it can bring to a patient* versus *just one person looking at a patient or the same person looking at that patient’s chart all the time.”* [Hud-7]

2) Reconfiguring roles and processes

Participants identified that “new information” about patients shared via HT reports and uploaded to the electronic medical record changed the flow of information in the clinic and created new opportunities for referrals and follow up. These processes allowed for a better understanding of individual patients’ health goals and risks and ways to address these, especially for medically complex patients. Aggregated data from reports provided huddle teams with increased knowledge about management of older adults’ most common health needs (e.g., nutrition, mobility, advance care planning) to inform clinical care, programming, and education.

Other aspects of the intervention, however, were viewed as less stable and sustainable. Participants suggested that greater role clarity was required between the huddle team and physicians, residents, and those outside the huddle, to facilitate the spread and effectiveness of team-based practices that were emerging in the clinic. Many participants wanted to see the intervention targeted toward socially and/or economically marginalized (elderly or otherwise) or more complex populations. Many participants expressed concern about resources required to sustain the volunteer component and the time and resources required to support inter-professional collaboration.

O*ne thing I wonder is whether TAPESTRY has managed to identify a healthier population than we need to be spending our time with. And as nice and creative and good customer service as it is for our patients, when we do have restricted resources, has the payoff been good enough for them and for us by selecting that population*. [PC-4].

As for other improvements, the process for referring patients into the program was seen as needing development as the intervention moves from a research program to usual practice.

## Discussion

This study used NPT to examine how an innovative primary care intervention aimed at older adults was implemented by an interdisciplinary team enhanced by trained volunteers. Using NPT increased our understanding about how providers and patients individually saw the HT intervention in comparison to usual care in general and in relation to teamwork (Coherence), how the team collectively bought into the new model of care (Cognitive Participation), how providers put the intervention into action (Collective Action), and how providers and patients appraised it (Reflexive Monitoring).

In relation to Coherence, participants in our study understood a purpose of the HT program was to strengthen interprofessional care delivery including volunteers as an expansion of the team as well as aiming to improve care coordination by the team. Their understanding of the intervention may be explained by HT clinical managers’ (i.e., lead physicians) who demonstrated support of the HT intervention through regular staff discussions in clinical rounds and other meetings. An integrated review of interdisciplinary working in primary care found that a barrier to collective teamwork was a physician training which is focused on the relationship of the doctor-patient dyad as well as the physicians’ overall sense of responsibility for patient care rather than sharing it with the team [23]. However, similar to our results, the review also found that having senior physicians or local champions facilitate interprofessional team working enabled buy-in for interdisciplinary work [23] and should be encouraged.

Under the construct of Cognitive Participation, huddle team members had a better understanding of each other’s roles and saw benefits in working as a team. Having dedicated meeting time to review patient charts and plan care for patient care in huddles appeared to contribute to this understanding. Fiscella and colleagues supported the notion of huddles to increase teamwork, as they found that these “teamlets” flourish when they have a shared cognitive model leading to an increased understanding of roles [28]. This requires trust, cooperation, communication, and feedback, resulting in better coordination of tasks. Case studies of innovative primary care practices in the Unites States also found that huddles helped team members coordinate tasks more efficiently [29]. Although huddles improved interdisciplinary practice, more work is needed to understand how to draw in team members outside of huddles.

The current study found that providers outside of the huddle team, namely physicians and medical residents, had difficulties understanding their roles in the HT intervention. This was mainly related to workflows which were not conducive to MDs (versus select MD champions) attending huddle meetings and ensuring that the huddle team regularly and effectively communicated with the MRPs regarding patient care planning. It appears that HT intervention developed a *provider huddle team* rather than the *patient’s team.* An effective *patient’s team* would need to consistently include the MRP for case conferencing, as well as the patient and/or their caregiver, and the volunteers. An effective *patient’s team* would engage all of these team members in care planning communication and follow up. Huddle teams were an effective strategy to get teams to work together; however, they were not necessarily effective in planning an individual’s care in the absence of all providers and family members who are members of the individual’s care team. Given the current focus for health systems to improve person-centred and more recently people-centered care [30], this was significant oversight in the HT intervention and requires enhancement in future HT implementation.

Researchers have found mixed levels of buy-in for interdisciplinary working by certain team members (i.e., physicians, pharmacists and nurses) with a tendency of physicians to resist collaborative teamwork, giving preference to working independently [23]. Primary care researchers explored contextual factors influencing team performance in five jurisdictions in three countries and found that despite moving to collaborative teams, primary care clinicians still tended to work in parallel, infrequently using team approaches to solve clinical problems [12]. They also found that team-based primary care was enhanced when direct physician involvement was not required in care provision. The huddle teams aimed to overcome these challenges with some success. However, similar to the challenges experienced by physicians and medical residents who were typically working outside of the huddle in our study, volunteers also largely worked on the periphery of the interprofessional team, expressing the desire to be more connected to the primary care team. Fully integrating lay community health workers into the primary care team has been shown to be a challenge by others [31]. To improve future HT implementation, strategies are needed to strengthen multi-directional communication and engagement among all primary care team members including physicians and volunteers.

Under the Collective Action construct, our results showed that there were a number of changes in team processes that occurred to support implementation of the HT intervention in relation to teamwork. These included changes in care coordination and case management processes, a shift to more proactive approaches in care provision by the team and refining patient information flow and content (e.g., more comprehensive patient information and focus on priorities based on patient’s goals). Based on field notes, these processes were refined over time requiring extensive teamwork. Researchers have found that establishing protocols can help team-work [23, 32]. One example in the current study was through the huddle’s creation of a decision path to support the triage process (Fig. 1). Despite these achievements, multi-directional information flow among team members needed improvement, which may have been strengthened through the development and full implementation of protocols to clarify roles and communication processes among all team members.

As seen in the current study, time and space resources were required to ensure team collaboration. This has long been supported by research that shows financial support for team activities has a positive effect on team performance [12, 23]. Despite the need for resources to support team activities, results showed that teams and patients perceived that HT was valuable, embedded well into the clinic environment, and worth maintaining. HT improved team communication within huddle teams and patients were satisfied overall with the intervention. Having a researcher (LC) attend huddle meetings and share her reflections with the team about implementation challenges supported reflective monitoring by the team. Given challenges in linking the providers with volunteers in the circle of care, there may be value in holding regular full team meetings to reflect on work processes and enhance role clarity. An international study found that a) outreach facilitators charged with driving change in primary care by “creating ‘peer pressure’, modelling good communication, encouraging reflection, supporting momentum and providing accountability” (p. 283) in the United States, b) facilitators who worked on quality assurance in Australia, and c) leadership training for practice leads and additional resources provided for clinical administration and meetings in Canada supported primary care reform processes that fostered team-oriented family practice [12]. These strategies may be valuable in spreading the HT model in other primary care settings.

This study had a number of strengths and limitations. Data was gathered from various sources (patients, providers, volunteers) and via various types (interviews, focus groups, and field observations) to support triangulation. Data was gathered prospectively over the course of the intervention. A robust coding strategy was developed over many meetings and reached agreement on final results within the large interprofessional research team. Primary care clinician scientists on the team supported peer debriefing and interpretation of results from a clinical vantage point. However, this could have introduced biases. The team attempted to prevent this by ensuring that a strong audit trail of decisions was maintained and having non-clinician researchers and research staff develop the initial themes supported by quotes. This intervention was tested in two sites of a Family Health Team in one urban area limiting generalizability.

## Conclusions

This study provides important insights about factors to consider when implementing interprofessional primary care team processes within a new model of care such as HT. The application of NPT constructs provides guidance for future implementation of primary care innovations involving interprofessional teams. Since this study was launched, NPT has become available as a Toolkit with a 23-item survey for use by teams implementing and evaluating complex interventions (http://www.normalizationprocess.org/nomad-study/). In this study, application of NPT constructs enriches our understanding of phases and timelines required for implementation of new approaches in care by interprofessional teams. Our data was collected at 4 months and 12 months into the implementation of a complex intervention, demonstrating that the normalization of new practices and processes were well underway. Given that timeframes for adoption of healthcare innovations may take years, this timeframe is relatively short to show evidence of normalization.

This study indicates that greater attention should be paid to the importance of the Cognitive Participation construct and focus on implementation strategies that would increase involvement of MRPs in interventions as a member of the *patient care* team as well as strengthening linkages of MRPs in *huddle teams* that may not directly involve them. This study also points to the important role of volunteers in primary care interventions, to suggest that these roles can also be integral to team-based and community-based care of older adults. A greater emphasis on clarity of roles, mechanisms for enhanced communication and strategies to ease workflow challenges should be carefully considered, planned, and implemented in normalizing primary care interventions among team members.

## Data Availability

The datasets analysed during the current study are not publicly available due to the lack of consent from participants to share the data beyond this study.

## Abbreviations

HT: Health TAPESTRY
MRP: Most responsible physician
NPT: Normalization process theory
